# Bacterial meningitis in diabetes patients: a population-based prospective study

**DOI:** 10.1038/srep36996

**Published:** 2016-11-15

**Authors:** Kiril E. B. van Veen, Matthijs C. Brouwer, Arie van der Ende, Diederik van de Beek

**Affiliations:** 1 Department of Neurology, Amserdam Neuroscience, Academic Medical Center, University of Amsterdam, Amsterdam, the Netherlands; 2 Department of Neurology, Medical Center Haaglanden, The Hague, the Netherlands; 3 The Netherlands Reference Laboratory for Bacterial Meningitis, Academic Medical Center, University of Amsterdam, Amsterdam, the Netherlands

## Abstract

Diabetes mellitus is associated with increased infection rates. We studied clinical features and outcome of community-acquired bacterial meningitis in diabetes patients. Patients were selected from a nationwide, prospective cohort on community-acquired bacterial meningitis performed from March 2006 to October 2014. Data on patient history, symptoms and signs on admission, treatment, and outcome were prospectively collected. A total of 183 of 1447 episodes (13%) occurred in diabetes patients. The incidence of bacterial meningitis in diabetes patients was 3.15 per 100,000 patients per year and the risk of acquiring bacterial meningitis was 2.2-fold higher for diabetes patients. *S. pneumoniae* was the causative organism in 139 of 183 episodes (76%) and *L. monocytogenes* in 11 of 183 episodes (6%). Outcome was unfavourable in 82 of 183 episodes (45%) and in 43 of 183 episodes (23%) the patient died. Diabetes was associated with death with an odds ratio of 1.63 (95% CI 1.12–2.37, P = 0.011), which remained after adjusting for known predictors of death in a multivariable analysis (OR 1.98 [95% CI 1.13–3.48], P = 0.017). In conclusion, diabetes is associated with a 2-fold higher risk of acquiring bacterial meningitis. Diabetes is a strong independent risk factor for death in community-acquired adult bacterial meningitis.

Diabetes mellitus type 1 and 2 have been associated with increased infection rates^1^, particularly bacterial infections^2^. Diabetes has been associated with a risk ratio of 1.21 for development of infection, and a risk ratio of 2.17 for hospitalization for infection^2^. Infections in diabetes patients are also more likely to be severe than in the non-diabetes persons^3^. Diabetes has been stated as risk for bacterial meningitis, in particular listeria meningitis although few studies have been done^4^,^5^,^6^,^7^,^8^,^9^,^10^. Retrospective studies have described diabetes to be associated with higher mortality rates among meningitis patients^5^,^6^. However, these studies were either retrospective and included only few patients^4^,^5^,^6^, or did not have diabetes as the focus of the article^7^,^8^,^9^,^10^. Therefore, little is known about bacterial meningitis in diabetes patients. We compared clinical features, outcome, and causative pathogens of community-acquired bacterial meningitis in patients with and without diabetes in a nationwide prospective cohort study.

## Methods

We conducted a nationwide, prospective cohort study on community-acquired bacterial meningitis. Methods have been described in detail previously^11^. Between March 2006 and October 2014, patients with bacterial meningitis over 16 years old were included. Bacterial meningitis was defined as a positive cerebrospinal fluid (CSF) culture or as a the combination of a positive blood culture with a relevant pathogen, or positive PCR or antigen test in cerebrospinal fluid for *Streptococcus pneumoniae* or *Neisseria meningitidis*, with at least one specific CSF finding predictive of bacterial meningitis consisting of a CSF leukocyte count >2000 cells/mm^3^, polymorphonuclear leukocyte count >1180 cells/mm^3^, glucose level <1.9 mmol/L, protein level >2 g/L, or CSF/blood glucose ratio <0.23^12^. Patients with a neurosurgical device, neurosurgical operation or procedure, and patients with neurotrauma within 1 month of the onset of meningitis were excluded from the cohort. Data on patient history, symptoms and signs on admission, laboratory findings, radiologic examination, treatment, and outcome were prospectively collected by means of a case record form (CRF). The CRF included a standard question on presence of diabetes in the medical history. In addition, all discharge letters were screened for a history of diabetes. We selected all patients reported to have diabetes by either the CRF or discharge letter. All patients underwent neurologic examination at hospital discharge, and outcome was graded using the Glasgow Outcome Scale. A favourable outcome was defined as a score of 5, and an unfavourable outcome was defined as a score of 1 to 4. Focal neurologic abnormalities were divided into focal cerebral deficits (e.g. aphasia, monoparesis, or hemiparesis) and cranial nerve palsies.

The criteria of the American Diabetes Association were used to define hypoglycaemia and hyperglycaemia^13^,^14^. A blood glucose level of <3.9 mmol/L (<70 mg/dL) was defined as hypoglycaemia, a non-fasting blood glucose levels of >7.8 mmol/L (>140 mg/dL) as hyperglycaemia, and a non-fasting blood glucose levels of >11.1 mmol/L (>200 mg/dL) as severe hyperglycaemia. Glycosylated haemoglobin (HbA1C) was not measured in the patients and no information on formal glucose tolerance testing was available.

The study period from January 2007 till January 2014 was used to determine the incidence of bacterial meningitis in the people with and without diabetes. The prevalence of diabetes in the Netherlands was obtained from the National Public Health Compass^15^, which estimated the point prevalence for January first, 2011. This prevalence was used to calculate the incidence of bacterial meningitis in diabetes patients during the study period. Incidence was calculated for all diabetic patients and with stratification according to age group (17–39, 40–64, >65 years old). Data on the general population was derived from Statics Netherlands (statline.cbs.nl).

### Ethical Statement

The MeninGene study was approved by the ethics committee of the Academic Medical Center, Amsterdam, The Netherlands. Ethical approval was granted in all participating centers and written informed consent was obtained from all participating individuals or legally authorized representatives. The study was conducted according to the principles of the Declaration of Helsinki (version of 2013, Fortaleza, Brazil) and in accordance with the Medical Research Involving Human Subjects Act (WMO) and other guidelines, regulations and acts.

### Statistics

Statistical analyses were performed with the use of SPSS statistical software, version 23 (SPSS Inc). For numerical and ordinal data the student t-test or Mann-Whitney U test were used. For categorical data the Fisher exact test was used. Logistic regression was used to examine the association between potential predictors and the likelihood of an unfavourable outcome and of death. Odds ratios (ORs) and 95% confidence intervals (CIs) were used to quantify the strength of these associations. Possible predictors of an unfavourable outcome and death were chosen on the basis of previous research^16^,^17^. The 95%-confidence interval (95%CI) for the incidence was calculated by using the Poisson distribution. All tests were 2-tailed, and p < 0.05 was considered significant.

## Results

A total of 1447 episodes of bacterial meningitis were included in the cohort between March 2006 and October 2014. A total of 183 episodes (13%) in 179 patients were reported to occur in diabetes patients. In 16 episodes no data on diabetes presence or absence could be obtained and these episodes were excluded from the analysis. Two diabetes patients had three episodes of pneumococcal meningitis during the study period of which one had a history of late onset agammaglobulinaemia next to diabetes.

In 2011, the estimated point prevalence of diabetes mellitus in the Netherlands was 834,000^15^. The incidence of bacterial meningitis in diabetes patients over 16-years old in the Netherlands was 3.15 per 100,000 patients per year (95% confidence incidence [CI] 2.73–3.64).

The incidence bacterial meningitis in persons over 16-years old without diabetes in the Netherlands was 1.44 per 100,000 persons per year (95% CI 1.36–1.52). The risk of acquiring bacterial meningitis was therefore 2.2-fold higher for diabetes patients (95% CI 1.9–2.6; P < 0.001).

The incidence of bacterial meningitis was 2.39 per 100,000 patients per year (95% CI 0.99–5.74) for diabetes patients between 17 and 40-years old, 3.43 (95% CI 2.76–4.28) for patients between 40 and 65-years old and 3.00 (95% CI 2.76–3.25) for patients over 65-years old. For non-diabetes persons the corresponding incidences were 0.70 (95% CI 0.61–0.79), 1.50 (95% CI 1.38–1.62) and 2.96 (95% CI 2.69–3.25) per 100,000 persons per year respectively. The risk of acquiring bacterial meningitis was 3.4-fold higher (95% CI 1.4–8.3; P = 0.007) for diabetes patients between 17 and 40-years old and 2.3-fold higher (95% CI 1.8–2.9; P < 0.001) for diabetes patients between 40 and 65-years old compared to their non-diabetes counterparts. No difference in risk was found for patients over 65-years old (1.0 [95% CI 0.13–1.26]; P = 0.903).

The median age of patients with diabetes and community-acquired bacterial meningitis was 65 years (range 25–91 years, Table 1). Type of diabetes mellitus was known for 87 episodes (48%), all of which were in type 2 diabetes patients. In 68 episodes (37%), patients used oral antidiabetic agents, in 16 episodes (9%) the combination of oral antidiabetic agents and insulin, and in 13 episodes (7%) insulin. Type of anti-diabetic agents was unknown in 86 episodes (47%). Conditions increasing the risk of bacterial meningitis, other than diabetes, were present in 44 of 183 episodes (24%) and consisted mostly of cancer (18 episodes), the use of immunosuppressive drugs (16 episodes), and alcoholism (12 episodes). Nine of these patients had more than one other predisposing factor. A distant focus of infection was present in 87 of 171 episodes (51%), with otitis or sinusitis (69 episodes; 38%) and pneumoniae (22 episodes; 13%) as most frequently encountered distant foci.

### Clinical features

Headache was present in 117 of 148 episodes (79%), and an altered mental state (defined by a Glasgow Coma Scale score below 14) was present on admission in 148 of 183 episodes (81%). Neck stiffness occurred in 125 of 173 episodes (72%) and fever (defined by a temperature of 38 °C or higher) in 144 of 181 episodes (80%). Symptoms and signs at presentation did not differ from non-diabetes bacterial meningitis patients (Table 2).

Blood chemistry tests showed diabetes patients had elevated whole blood leukocyte counts (median 16.3 × 10^9^/L [interquartile range (IQR) 11.6–22.6 × 10^9^/L]) and C-reactive protein was elevated (>8.0 mmol/L) in 169 of 173 episodes (98%; median 172 mg/L [IQR 71–298 mg/L]). Leukocyte count and C-reactive protein levels did not differ between patients with and without diabetes. Upon presentation hyperglycaemia was present in 158 of 175 episodes (90%). Median blood glucose level was 12.6 mmol/L (IQR 10.2–16.0 mmol/L). Severe hyperglycaemia was more often present in diabetes patients compared to patients without diabetes (118 of 175 [67%] vs. 256 of 1191 [21%], P < 0.001).

A lumbar puncture was performed in all patients. Independent predictors of bacterial meningitis^12^ were present in 160 of 183 episodes (87%). Median CSF leukocyte count was 3,300 cells/mm^3^ (IQR 1,006–9,860 cells/mm^3^) and in 44 of 177 episodes (25%) CSF leukocyte count was below 1,000 cells/mm^3^.

Neuroimaging (computed tomography) was performed on admission in 165 of 183 episodes (90%). In 50 of 165 episodes (30%) imaging showed signs of sinusitis or otitis, in seven of 156 episodes (4%) generalized brain oedema, in five of 155 episodes (3%) hydrocephalus and in six of 155 (4%) a hypodense lesion consistent with cerebral infarction was found.

*S. pneumoniae* was the causative organism in 139 of 183 episodes (76%), *L. monocytogenes* in 11 of 183 episodes (6%), and *N. meningitidis* in 10 of 183 episodes (5%; Table 1). Meningococcal meningitis was less often encountered in diabetes patients as compared to the non-diabetes meningitis patients (10 of 183 [5%] vs. 137 of 1248 [11%]; P = 0.019). *Pseudomonas aeruginosa* (in two patients) and *Nocardia farcinica* (in one patient) were identified only in diabetes patients.

### Complications and outcome

Neurological complications during admission in diabetes patients consisted of seizures in 28 of 172 episodes (16%), cerebrovascular accidents in 23 of 169 episodes (14%), hydrocephalus in 5 of 164 episodes (3%) and sinus thrombosis in 2 of 162 episodes (1%). Neurological complications did not differ between patients with and without diabetes.

Cardiorespiratory failure occurred in 84 of 179 episodes (47%) and was more common in diabetes patients as compared to the patients without diabetes (84 of 179 [47%] vs. 453 of 1212 [37%]; P = 0.017). Admission to the intensive care unit was similar in patients with and without diabetes (98 of 183 [54%] vs. 615 of 1248 [49%], P = 0.304), but diabetes patients more often had respiratory failure (57 of 176 [32%] vs. 300 of 1204 [25%], P = 0.042) and need of mechanical ventilation (71 of 176 [40%] vs. 391 of 1197 [33%], P = 0.049) compared to patients without diabetes.

Outcome was unfavourable in 82 of 183 episodes (45%) of bacterial meningitis in diabetes patients and in 43 of 183 episodes (23%) the patient died. In univariable analysis diabetes mellitus was associated with death with an OR of 1.63 (95% CI 1.12–2.37, P = 0.011; Table 3). The effect size of diabetes for death remained after adjusting for known predictors of death (age, otitis and sinusitis, neck stiffness, score on GCS, blood thrombocyte count, CRP, CSF leukocyte count, CSF protein, *L.* monocytogenes, *S. pneumoniae*), blood glucose and treatment with adjunctive dexamethasone in a multivariable analysis (OR 1.98 [95% CI 1.13–3.48], P = 0.017).

### Antimicrobial and adjunctive dexamethasone therapy

Initial antimicrobial therapy was reported for 180 of 183 episodes (98%) and included a combination of amoxicillin or penicillin and third-generation cephalosporin in 99 episodes (55%), monotherapy third-generation cephalosporin in 42 episodes (23%), monotherapy amoxicillin or penicillin in 37 episodes (21%), and other in two episodes (1%). Inadequate initial antimicrobial therapy was started in four listerial meningitis episodes consisting of monotherapy with a third-generation cephalosporin. In seven episodes patients (4%) were treated additionally with acyclovir on the suspicion of viral encephalitis.

Adjunctive dexamethasone in diabetes patients was initiated before or together with the antibiotics in 139 of 181 episodes (77%) and in 134 of 181 episodes (74%) given according to guideline recommendations (10 mg four times per day for 4 days, administered before or together with the antibiotics)^18^. Fewer diabetes patients with meningitis received dexamethasone compared to non-diabetes meningitis patients (139 of 181 [77%] vs. 1095 of 1203 [91%]; p < 0.001). Unfavourable outcome was observed in 57 of 136 episodes (42%) of patients treated with adjunctive dexamethasone and 29 of 136 patients (21%) died. Outcome (favourable or unfavourable) and mortality did not differ between diabetes patients treated with and without dexamethasone (79 of 136 [58%] vs. 20 of 45 [44%], P = 0.123 and 29 of 136 [21%] vs. 14 of 45 [31%], P = 0.225). During dexamethasone treatment hyperglycaemia requiring insulin was reported in 17 episodes (13%). One gastro-intestinal bleeding was reported after the use of dexamethasone, but despite the bleeding the patient had a good recovery.

## Discussion

Diabetes patients are at 2-fold higher risk for bacterial meningitis as compared than non-diabetes patients. This is in line with previous retrospective studies describing diabetes as a risk factor for bacterial infections^1^,^2^. Our study was prospective and performed nationwide in the Netherlands (population, 16 million). Diabetes may lead to an immunocompromised state by decreased efficacy of cell-mediated immunity, with relatively malfunction of polymorphonuclear leukocytes, monocytes and lymphocytes, which have all been associated with degree and duration of hyperglycaemia in patients^3^,^19^. We also found that diabetes is an independent predictor of mortality in bacterial meningitis. This effect could not be explained by high blood glucose levels, which has previously been related to poor outcome^20^.

The distributions of pathogens between diabetes and non-diabetes patients were similar. Most common cause among diabetes patients was *S. pneumoniae* (76%), followed by *L. monocytogenes* (6%) Diabetes patients have previously been described to be at particular high risk for *listeria* meningitis. Our data show that at least in Netherlands this is not the case. Some uncommon pathogens, such as *Pseudomonas aeruginosa* and *Nocardia farcinica*, were identified in diabetes patients only. Our findings are in contrast to studies from Korea and Taiwan, describing *K. pneumoniae* as causative pathogen in two-thirds of diabetes patients with meningitis^4^,^8^,^10^. However, this can be explained by differences in general epidemiology of infectious diseases between the continents^21^. As *L. monocytogenes* is the second most common causative organism in diabetes patients identified in our cohort, empiric treatment for diabetes patients must include ampicillin or amoxicillin to cover listeria^22^,^23^.

Diabetes patients were less likely to be treated with adjunctive dexamethasone therapy. Physicians may be hesitant to administer corticosteroids in diabetes patients. First, administration of corticosteroids in diabetes patients may cause dysregulation of diabetes. This did occur, but only in 13% of diabetes patients on dexamethasone. Although hyperglycaemia has been associated with unfavourable outcome in bacterial meningitis^7^, this detrimental effect probably does not counterbalance the favourable effect of adjunctive dexamethasone in pneumococcal meningitis^24^,^25^,^26^,^27^. Second, patients with diabetes have been described to be at risk for atypical pathogens. The effect of adjunctive dexamethasone therapy was mainly driven by the effect in the pneumococcal subgroup^24^,^25^,^26^,^27^. The current study showed that causative pathogens between diabetes and non-diabetes meningitis patients are similar. Guidelines recommend starting 10 mg of dexamethasone four times per day for four days, administered before or together with the antibiotics, in bacterial meningitis patients, including those with diabetes^18^,^25^,^28^. In non-pneumococcal bacterial meningitis patients with and without diabetes, dexamethasone should be carefully considered on an individual patient basis.

Our study has several limitations. The observational design of the study is sensitive to the introduction of confounding factors, which hinder the evaluation of the risk analysis. Although the incidence of diabetes mellitus in the Netherlands was estimated, this was done by a reliable source. Estimation was made by the National Public Health Compass, which is a part of the National Institute of Public Health and the Environment, and was based on data provided by the Netherlands Institute for Health Services Research to which most general practices are connected. Some specific information on diabetes patients was collected retrospectively, e.g. type of diabetes, diabetes treatment, and complications of the use of dexamethasone. This might lead to an underestimation of the complications associated with dexamethasone. Other limitations of this study were that only patients with culture-proven meningitis were included in our cohort study. Not all patients with suspected bacterial meningitis may undergo a lumbar puncture, e.g. patients with coagulopathy due to sepsis or those with space-occupying lesions on cranial imaging. Signs of sepsis or mass effect on cerebral CT are well defined predictors for unfavourable outcome^16^,^29^. Underrepresentation of these patients in our cohort, may lead to an underestimated of unfavourable outcome and mortality.

## Additional Information

**How to cite this article**: van Veen, K. E. B. *et al.* Bacterial meningitis in diabetes patients: a population-based prospective study. *Sci. Rep.* **6**, 36996; doi: 10.1038/srep36996 (2016).

**Publisher’s note**: Springer Nature remains neutral with regard to jurisdictional claims in published maps and institutional affiliations.

## Figures and Tables

**Table 1:** Characteristics of diabetes patients with bacterial meningitis^a^.

Characteristic	n/N (%)	Characteristic	n/N (%)
Age (years)	65 (25–91)	Blood chemistry tests^d^	
Female	85/183 (46)	Leukocyte count (x10^9^/L)	16.3 (2.5–41.9)
Diabetes mellitus type 2	87/183 (48)	Thrombocytes (x10^12^/L)	208 (39–868)
Type unknown/not reported	96/183 (52)	C-reactive protein (mg/L)	172 (4–736)
Predisposing conditions^b^	44/183 (24)	Glucose (mmol/L)	12.6 (1.5–30.9)
Cancer	18/181 (10)	Indexes of inflammation in CSF^e^	
Immunosuppressive drugs	16/182 (9)	Leukocyte count (cells/mm^3^)	3,300 (1–75,000)
Alcoholism	12/166 (7)	Protein (g/L)	4.31 (0.44–19.70)
Distant foci of infection	87/171 (51)	CSF/blood glucose ratio	0.16 (0.0–1.55)
Otitis media or sinusitis	69/182 (38)	Causative organism	
Pneumonia	22/170 (13)	*Streptococcus pneumoniae*	139/183 (76)
Symptoms and signs on admission		*Listeria monocytogenes*	11/183 (6)
Duration of symptoms >24 h	88/171 (51)	*Neisseria meningitidis*	10/183 (5)
Headache	117/148 (79)	*Escherichia coli*	4/183 (2)
Nausea	81/140 (58)	*Streptococcus species*^f^	10/183 (5)
Temperature ≥38 °C	144/181 (80)	*Staphylococcus aureus*	2/183 (1)
Neck stiffness	125/173 (72)	*Staphylococcus epidermidis*	1/183 (1)
Triad^c^	86/173 (50)	*Haemophilus influenza*	2/183 (1)
Signs of septic shock	56/177 (32)	*Pseudomonas aeruginosa*	2/183 (1)
Seizures	8/170 (5)	*Nocardia farcinica*	1/183 (1)
Altered mental state (EMV <14)	148/183 (81)	*Klebsiella pneumoniae*	1/183 (1)
Coma (EMV <8)	30/183 (16)	Outcome	
Focal neurological deficits	57/181 (31)	Unfavorable outcome	82/183 (45)
Cranial nerve palsy	15/154 (10)	Mortality	43/183 (23)
Brain imaging	165/183 (90)	Neurological sequelae	49/122 (40)
Abnormal	65/165 (39)	Hearing loss	16/124 (13)
Otitis or sinusitis on CT/MRI	50/165 (30)		

CSF: cerebrospinal fluid.

^a^Data are presented as n/N (%), or median (range).

^b^Not directly related to diabetes mellitus.

^c^Triad of fever, neck stiffness, and change in mental status.

^d^Leukocyte count was known in 179 episodes, C-reactive protein in 173 episodes, glucose in 175 episodes and thrombocyte count in 176 episodes.

^e^CSF Leukocyte count was known in 177 episodes, CSF protein levels in 170 episodes, CSF blood to glucose ratio in 169 episodes.

^f^4 Streptococcus agalactiae, 2 Streptococcus pyogenes, 1 Streptococcus gallolyticus, 1 Streptococcus anginosus, 1 Streptococcus mitis, 1 Streptococcus salivarius.

**Table 2:** Comparison between patients with and without diabetes mellitus^a^.

Characteristic	Diabetes mellitus +	Diabetes mellitus −	P-value
Age (years)	65 (25–91)	60 (17–94)	**<0.001**
Female	85/183 (46)	617/1248 (49)	0.477
Symptoms and signs on admission			
Headache	117/148 (79)	899/1089 (83)	0.304
Neck stiffness	125/173 (72)	868/1167 (74)	0.577
Temperature ≥38 °C	144/181 (80)	905/1228 (74)	0.100
Triad of fever, neck stiffness, and change in mental status	86/173 (50)	489/1190 (41)	**0.039**
Predisposing factors^b^	44/183 (24)	306/1247 (25)	0.927
Distant focus of infection	87/171 (51)	520/1201 (43)	0.070
Blood chemistry tests			
Leukocyte count (x10^9^/L)	16.3 (2.5–41.9)	17.0 (0.1–99.8)	0.659
Thrombocytes (x10^12^/L)	208 (39–868)	198 (0–955)	**0.005**
C-reactive protein (mg/L)	172 (4–736)	192 (0–752)	0.336
Glucose (mmol/L)	12.6 (1.5–30.9)	8.8 (0.1–31.3)	**<0.001**
Indexes of inflammation in CSF			
Leukocyte count (cells/mm3)	3,300 (1–75,000)	2,315 (0–463,149)	**0.005**
Leukocyte count <1000 cells/mm^3^	44/177 (25)	390/1136 (34)	**0.013**
Independent CSF predictors present	160/183 (87)	1071/1248 (86)	0.648
CSF/blood glucose ratio	0.16 (0.00–1.55)	0.03 (0.00–1.67)	**<0.001**
Causative organism			
*Streptococcus pneumoniae*	139/183 (76)	876/1248 (70)	0.117
*Neisseria meningitidis*	10/183 (5)	137/1248 (11)	**0.019**
*Listeria monocytogenes*	11/183 (6)	68/1248 (5)	0.729
Outcome			
Unfavorable outcome	82/183 (45)	449/1248 (36)	**0.022**
Mortality	43/183 (23)	198/1248 (16)	**0.015**
Neurological sequelae	49/122 (40)	326/925 (35)	0.315
Hearing loss	16/124 (13)	118/1047 (11)	0.553

CSF: cerebrospinal fluid. P values < 0.05 are giving in bold.

^a^Data are presented as n/N (%), or median (range).

^b^Other than immunosuppressive drugs.

**Table 3:** Univariable and multivariable analyses in patients with bacterial meningitis on risk factors for death.

Variable	Univariable analysis		Multivariable analysis	
	OR (95% CI)	P value	OR (95% CI)	P value
Diabetes mellitus	1.63 (1.12–2.37)	**0.011**	1.98 (1.13–3.48)	**0.017**
Blood glucose				
Normal (3.9–7.8 mmol/L)	Reference		Reference	
Low (<3.9 mmol/L)	2.22 (0.40–12.35)	0.363	0.43 (0.06–3.08)	0.430
High (>7.8–11, 1 mmol/L)	0.67 (0.47–0.95)	**0.023**	0.64 (0.40–1.05)	0.076
Severe high (>11, 1 mmol/L)	1.09 (0.76–1.58)	0.640	0.84 (0.47–1.48)	0.538
Adjunctive dexamethasone	0.40 (0.30–0.55)	**<0.001**	0.62 (0.40–0.97)	**0.035**
Age	1.05 (1.04–1.06)	**<0.001**	1.04 (1.02–1.05)	**<0.001**
Otitis or sinusitis	0.40 (0.29–0.57)	**<0.001**	0.58 (0.37–0.93)	**0.022**
Neck stiffness	0.50 (0.37–0.69)	**<0.001**	0.51 (0.34–0.78)	**0.002**
Score on GCS	0.84 (0.80–0.88)	**<0.001**	0.85 (0.79–0.90)	**<0.001**
Blood thrombocyte count				
<150 × 10^12^/L	2.18 (1.60–2.97)	**<0.001**	1.35 (0.87–2.08)	0.178
150–450 × 10^12^/L	Reference		Reference	
>450 × 10^12^/L	2.84 (1.21–6.66)	**0.016**	2.32 (0.56–9.66)	0.247
CRP (per 10 mg/l)	1.04 (1.03–1.05)	**<0.001**	1.04 (1.02–1.05)	**<0.001**
CSF leukocyte count				
<100 cells/mm^3^	Reference			
100–999 cells/mm^3^	0.48 (0.31–0.74)	**0.001**	0.58 (0.32–1.04)	**0.067**
1000–10,000 cells/mm^3^	0.19 (0.12–0.29)	**<0.001**	0.28 (0.15–0.51)	**<0.001**
>10,000 cells/mm^3^	0.20 (0.12–0.34)	**<0.001**	0.25 (0.12–0.53)	**<0.001**
CSF protein (g/L)	1.09 (1.05–1.13)	**<0.001**	1.09 (1.04–1.15)	**0.001**
*Listeria monocytogenes*	2.76 (1.70–4.50)	**<0.001**	1.99 (0.85–4.64)	0.114
*Streptococcus pneumoniae*	1.03 (0.76–1.39)	0.869	0.87 (0.51–1.49)	0.617

CRP: C-reactive protein, CSF: cerebrospinal fluid, GCS: Glasgow coma scale.

P values < 0.05 are giving in bold.
